# Molecular identification of methane-consuming bacteria in the Persian Gulf: a study for microbial gas exploration

**DOI:** 10.3389/fmicb.2024.1435037

**Published:** 2024-11-08

**Authors:** Mahsa Harirforoush, Mahmoud Shavandi, Mohammad Ali Amoozegar, Parvaneh Saffarian, Shabnam Hasrak

**Affiliations:** ^1^Department of Biotechnology, Science and Research Branch, Islamic Azad University, Tehran, Iran; ^2^Microbiology and Biotechnology Group, Environment and Biotechnology Research Division, Research Institute of Petroleum Industry, Tehran, Iran; ^3^Extremophiles Laboratory, Department of Microbiology, School of Biology, College of Science, University of Tehran, Tehran, Iran; ^4^Genome Center, National Institute of Genetic Engineering and Biotechnology, Tehran, Iran

**Keywords:** microbial prospecting, methanotrophs, NGS, biodiversity, sediments of the Persian Gulf, gas reserves

## Abstract

The seepage of gaseous compounds from underground reservoirs towards the surface causes abnormalities in the population of microbial communities that consume light hydrocarbons on the surface of the reservoir. This microbial population can serve as indicators for determining the location of gas reservoirs prior to drilling operations. In this study, the simulation of methane gas leakage in the sediments of the Persian Gulf was conducted using a laboratory model. The objective of this simulation was to identify the microbial population consuming methane within the sediments of the Persian Gulf, aiding in the exploration of gas reserves. Continuous injection of methane gas into the system was performed for a period of 3 months to enrich the microbial consortia consuming methane. Subsequently, the microbial population was identified using next-generation sequencing (NGS) analysis. The results indicated that, based on the 16S rRNA sequencing dataset, aerobic methanotrophs, including genera *Methylobacter*, *Methylomarinum*, *Methylomicrobium, Methylomonas*, and *Methylophage*, were the dominant microbial group on the surface of the sediments. Additionally, anaerobic methane oxidation archaea in sediments were performed by ANME-2 and ANME-3 clades. The findings demonstrate that these microbial communities are capable of coexistence and thrive in long-term exposure to methane in the sediments of the Persian Gulf. Identifying this microbial pattern, alongside other geophysical and geological data, can increase the success rate of gas reservoir exploration.

## Highlights

- Laboratory simulation of methane gas leakage in Persian Gulf sediments.
- Identification of key microbial groups: aerobic methanotrophs and anaerobic methane-oxidizing archaea.
- Microbial indicators enhance gas reservoir exploration success.

## Introduction

The Microbial Prospecting for Oil and Gas (MPOG^®^) is a renowned approach to petroleum exploration. The method relies on the upward seepage of light hydrocarbon gases from underground reservoirs to the earth’s surface. In this technique, hydrocarbon-oxidizing bacteria present in surface soils or sediments utilize these gases, including methane, ethane, propane, and butane, as a carbon source to fuel their metabolic activities and growth. The presence of hydrocarbon-degrading microorganisms can be considered as an indirect proxy for the occurrence of light hydrocarbons, implying that their detection could serve as a valuable indicator for the presence of oil and gas deposits in the subsurface (MicroPro GmbH Microbiological Laboratories Gommern, n.d.; Rasheed et al., 2014).

MPOG^®^ has been demonstrated to be a highly effective method with a success rate of 90%. When combined with geological, geochemical, and geophysical methods, this technique can provide a comprehensive evaluation of the hydrocarbon potential of an area, allowing for more informed decision-making regarding drilling locations. By reducing drilling risks and increasing the chances of success in petroleum exploration, this approach has the potential to greatly benefit the industry (Rasheed et al., 2013).

By implementing novel techniques to distinguish the activities of methane-oxidizing bacteria (methanotrophs), it becomes feasible to differentiate between oil fields with a free gas cap and those without, as well as pure gas fields. These bacteria serve as reliable indicators of gas presence in pure gas fields, while those that solely oxidize ethane and higher hydrocarbons indicate oil fields. Methanotrophs utilize methane as their primary source of carbon and energy for both anabolic and catabolic processes, using it as a reduced carbon substrate (Taubert, 2019). These bacteria are typically the most abundant hydrocarbon-oxidizing bacteria populations found in gas fields, primarily because methane is the predominant component of light hydrocarbons in oil and gas reservoirs (Zhang et al., 2017; Rasheed et al., 2014). These advancements in analysis offer valuable insights into subsurface hydrocarbon reservoirs and aid to the development of effective oil exploration strategies (Wagner et al., 2002).

Phylogenetically, aerobic methanotrophs are known to belong to three different phyla, namely *Proteobacteria*, *Verrucomicrobia*, and the candidate division NC10 (Islam et al., 2020; Hakobyan and Liesack, 2020; Altshuler et al., 2022). Within the phylum *Proteobacteria*, methanotrophs are classified into three categories based on their 16S ribosomal RNA (rRNA) phylogeny, Type I, Type II, and Type X methanotrophs are classified, respectively, as members of the *Gammaproteobacteria*, *Alphaproteobacteria*, and intermediate Type X methanotrophs belong to the *Gammaproteobacteria* (Roldán et al., 2022). *Verrucomicrobia* are extremely acidophilic methanotrophs and include the genera *Methylacidimicrobium* and *Methylacidiphilum* (Kalyuzhnaya and Xing, 2018). The NC10 phylum’s representative bacterium, known as *‘Candidatus Methylomirabilis oxyfera’* (*M. oxyfera*), has the ability to perform aerobic methane oxidation under anaerobic conditions by utilizing oxygen produced from nitric oxide (He et al., 2016; Raghoebarsing et al., 2006; Ettwig et al., 2009).

Anaerobic methane oxidation (AOM) is a critical global phenomenon executed by microorganisms, which plays a vital role in limiting the discharge of a considerable amount of methane into the atmosphere (Knittel and Boetius, 2009). The biological mechanisms underlying AOM remain poorly understood, primarily due to the challenges associated with cultivating anaerobic methanotrophic archaea (ANME) and the microorganisms that interact with them. To date, no microorganism capable of performing AOM has been successfully isolated in a pure culture, which presents a significant hurdle for researchers. ANME inhabit a wide range of environments, and molecular techniques have demonstrated their presence in almost all ecosystems associated with methane emissions (Slobodkin et al., 2023).

Various ANME groups have the ability to perform anaerobic methane oxidation while reducing different electron acceptors, and the ultimate step of reduction can occur either within the ANME cells or through extracellular electron transfer with a bacterial partner. It has been well-established that AOM can be coupled with the reduction of sulfate, nitrate, and nitrite, as well as Fe(III) and Mn(IV) compounds (Lu et al., 2016; Luo et al., 2018; Luo et al., 2019). The process of anaerobic oxidation of methane (AOM) is mediated by syntrophic aggregates of ANME and sulfate-reducing bacteria (SRB) from the Deltaproteobacteria (Cui et al., 2015). ANME lineages are diverse and include ANME-1a, −1b, −2ab, −2c, and − 3, as well as lineages that appear to be geographically restricted. These microorganisms have been identified in seep sediments around the world. This coupling of methane oxidation and sulfate reduction is a key process in these ecosystems. Deltaproteobacteria members of AOM consortia have also been discovered, including the SEEP SRB1a cluster from the *Desulfosarcina/Desulfococcus* group, some *Desulfobulbus* lineages, SEEP SRB2 from hydrothermal systems, and *“Candidatus Desulfofervidus*”/hot seep 1. These microorganisms play an essential role in the biogeochemical cycling of methane in various environments, highlighting the importance of understanding their distribution, diversity, and metabolic capabilities (Vigneron et al., 2019; Yu et al., 2022). SEEP-SRB1, is the most common bacterium associated with ANME-1 and ANME-2 in natural and laboratory AOM sulfate-reducing consortia. ANME-2, on the other hand, is frequently linked with SEEP-SRB2, which belongs to *Desulfobacterales*, while ANME-3 tends to be associated with bacteria related to *Desulfobulbus* (Yu et al., 2022).

The Persian Gulf region contains an immense amount of petroleum, with its reserves accounting for 57% of the world’s oil reserves and 46% of natural gas reserves. As such, it serves as the primary source of fossil energy on a global scale. One of the key reasons for the region’s importance in the global energy market is the abundance of its petroleum reserves. This area is one of the most actively explored regions of the world, containing a large number of oil and gas fields (Rabbani, 2007).

In this study, a laboratory model was used to simulate methane gas leakage in the sediments of the Persian Gulf. The method employed the MPOG® technique, which facilitates methane migration on the sediment surface. Through continuous injection of methane into the model, methane-oxidizing bacteria were enriched on the sediment surface. By analyzing the microbial population using next-generation sequencing (NGS), we obtained a pattern of microorganisms that consume methane. This pattern can be utilized to mitigate drilling risks and assist in the exploration of gas reservoirs.

## Materials and methods

### Sampling and microcosm simulation

To simulate methane gas leakage in the sediments of the Persian Gulf, an area with no previous history of methane seepage was selected in May 2021. Water and sediment samples were obtained from the northwestern region of the Persian Gulf (Nayland Gulf), (27.431088 N, 52.643542 E), using a Niskin bottle and a grab sampling device, respectively. The sediment samples were specifically collected from a depth of 20 meters. The sediment samples were stored in sterilized bags at 4°C prior to the commencement of the experimental stages.

**Figure 1:**
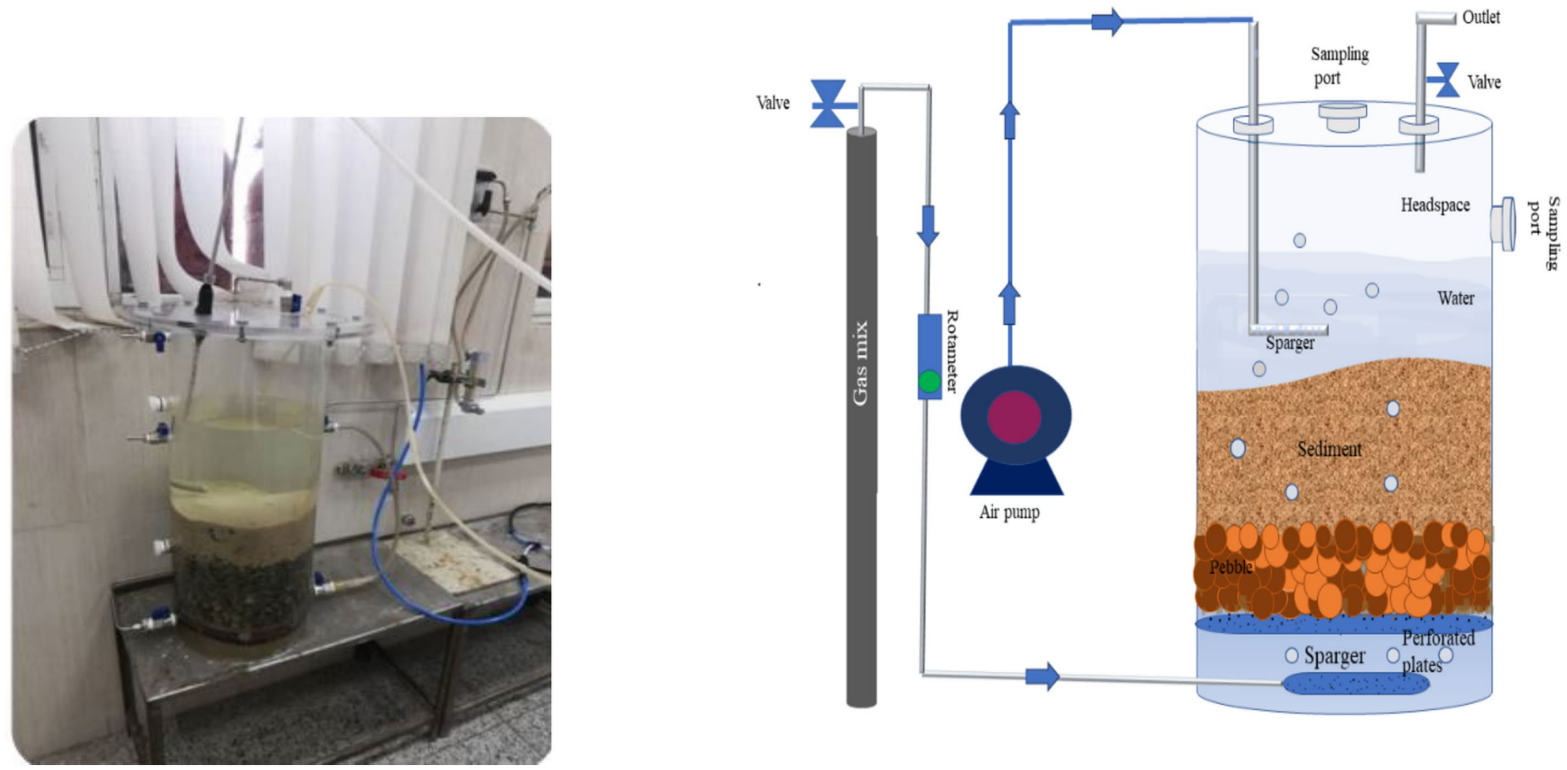
Schematic representation and photograph of the microcosm.

As illustrated in Figure 1, a microcosm was constructed from Plexiglas with a height of 60 cm and an inner diameter of 25 cm. Perforated plates were located at the base of the microcosm and a layer of pebbles (10 cm) was placed on top of these plates to prevent the clogging of the screen pores by sediments and to ensure the uniform distribution of methane gas in sediment layer. Above the pebbles, the microcosm was inoculated with 15 cm of sediments, followed by 30 cm of fresh water, leaving 10 cm of headspace. Two ports for water and sediment sampling were drilled along the microcosm. Since light prevents methane oxidation, the system was completely covered with aluminum foil.

In order to simulate the CH_4_ and air migration to the surface in this model, a mixture of CH_4_ gas and air was continuously sparged into the base of the microcosm. Due to the high flammability of methane in the presence of air, a gas blend with a volume ratio of 2.5% methane and 97.5% air has been utilized to ensure the safety of the system. The flow rate of the gas mixture in the inlet was maintained at a fixed rate of 5 mL/min for a period of 3 months, which was controlled by glass rotor flowmeters.

As the dissolved oxygen (DO) concentration in the sampling area was measured to be approximately 4.45 mg/L, a sparger was employed to ensure a constant DO level on the sediment surface. Concurrently, DO and pH variations were monitored by HQ40d multi-meter (HACH) throughout the experiment. The concentration of nitrate (NO_3_^−^), phosphate (PO_4_), and sulphate (SO_4_^−2^) in the water of the system was measured monthly. After 90 days of continuous methane injection into the microcosm, gas chromatography was employed to measure the outlet gas concentrations using an Agilent gas chromatography machine, model 7890B.

### Total heterotrophic microbial count

The Standard Plate Count (SPC) method was utilized to quantify the total number of heterotrophic microbial present in water and sediment samples obtained from microcosm every week. R2A agar medium was used as a growth medium for this purpose. In order to determine the number of culturable bacteria, total bacterial count was performed on R2A agar using the serial dilution method in triplicate. The cultured plates were then incubated at 25°C for 3 days (Gibbs and Hayes, 1988).

### Enumeration of methane-oxidizing bacteria

In this study, the most probable number (MPN) method was employed to enumerate Methane-Oxidizing Bacteria from sediment samples. To achieve this, 10 serum bottles were prepared, each containing 9 mL of Nitrate Mineral Salt (NMS) medium (ATCC^®^ MD-1306). One gram of sediment sample was then dissolved in 20 mL of 0.3% normal saline solution, and the resulting suspension was incubated for 1 h with shaking at 25°C, followed by a resting period of 30 min. Serial dilutions were performed on the upper sediment suspension (ranging from 10^1−^ to 10^10−^), with the addition of 1 mL of this suspension to the serum bottles containing NMS medium. To prevent fungal contamination, 0.03 mg/mL of cycloheximide was added to all serum bottles. All serum bottles were then incubated anaerobically using a controlled leakage anaerobic jar filled with a mixture of 50% methane and 50% air. The jar was wrapped with aluminum foil and placed in a lab shaker incubator at 25°C for 2 weeks. Positive cultures were identified by the visible turbidity in the serum bottles.

Negative controls were employed to validate the experimental setup, specifically, NMS medium without a carbon source and NMS medium containing the composition of air and methane without sediment suspension. These negative controls ensured that any observed growth of Methane-Oxidizing Bacteria resulted from the presence of sediment samples and not external factors like media or incubation condition. The use of negative controls in microbial ecology studies is critical to ensure the accuracy and reliability of the results (Rahalkar et al., 2021; Rusmana and Akhdiya, 2009).

### Isolation of methane-oxidizing bacteria, DNA extraction, and phylogeny identification

The Isolation of Methane-Oxidizing Bacteria from the sediment of the microcosm was performed using the serum bottles from the previous stage in which the bacteria had grown. Firstly, the bacteria needed to be transferred from the liquid culture medium to the solid medium. To prepare the solid NMS medium in the plate, 1.5% (w/v) agar was added to the basic NMS salt medium. Then, under sterile conditions, 100 μL of the serum bottle contents were inoculated onto the surface of the solid medium in the plate. Methane-utilizing colonies were obtained by incubating NMS agar plates for 2 weeks at 25°C inside a gas-tight jar with the gas composition described above. After successive cultivation on a specialized medium, single colonies were eventually obtained (Rahalkar et al., 2021).

The genomic DNA was extracted from isolated bacteria colony by PGA Bacterial DNA Extraction Kit (PouyaGene- Iran). To identify the phylogenetic relationship among methane-consuming strains, PCR amplification of the 16S rRNA gene using universal primers (27F and 1492R) was performed, and the PCR products were sent for sequencing (Novogene, Hong Kong). Subsequently, the closest microorganism to each strain was identified by comparing its 16S rRNA gene sequence with the sequences available in the EzTaxon server.

### PCR amplification of the pmoA gene

In this study, the presence of the *pmoA* gene, which encodes the key enzyme pMMO in the bacterial methane metabolism pathway, was utilized for the authentication of bacterial isolates. Detection of the gene in the isolates was achieved through partial amplification using specific primers. The primer set A189/mb661 was utilized to perform PCR amplification of the *pmoA* gene in methanotrophic bacteria (Jhala et al., 2014; Altshuler et al., 2022). The PCR reaction was performed as follows: initial denaturation at 95°C for 5 min, followed by 22 cycles of denaturation at 94°C for 45 s, annealing at 56°C for 60 s, extension at 72°C for 60 s, and a final extension at 72°C for 10 min (Liu et al., 2016).

### DNA extraction of sediments

To investigate the microbial population before and after methane injection, DNA extraction was carried out from the original sediment (OS) and the sediment present in the microcosm (MS) from the top 0–5 cm sediment layer, which was continuously exposed to methane injection for 3 months. The DNA extraction from sediment samples was performed using the DNeasy PowerMax Soil DNA Extraction Kit (QIAGEN 12988–10, Germany) following the manufacturer’s instructions. Extracted DNA samples were sequenced using Illumina Novaseq 6,000 platform (PE150) (Novogene, Hong Kong).

### Ribosomal RNA classification

Initially, to improve the quality of the data obtained from a sequencing experiment, the bbduk.sh tool, which is a part of the bbmap toolkit software, was utilized. This tool effectively filtered out reads with low quality scores, using a Phred quality score threshold of 18. Then for each dataset, a sample of 5 million reads was extracted and analyzed to identify those corresponding to ribosomal RNA genes. This was achieved using SSU-ALIGN (Nawrocki, 2009). The resulting putative prokaryotic 16S rRNA gene sequences were then compared to the SILVA reference database (release 138.1 SSUParc) using BLAST. Taxonomic classification was assigned based on the closest match with a similarity threshold of ≥90%, provided the read was at least 90 base pairs in length. These steps were taken as part of the data analysis process for the study. Finally, the abundance of each taxon present in the sediment samples was obtained at different phylogenetic levels. The presence of various taxa at the phylum, order, and class level, and also their abundance in each sample were visualized (Rezaei Somee et al., 2021).

### Scanning electron microscopy (SEM) and confocal laser scanning microscopy (CLSM) analysis

After 90 days of continuous methane injection into the microcosm, microbial biofilm formation on the surface of the sediment was observed using scanning electron microscopy (SEM) (VEGA\\TESCAN-XMU) and confocal laser scanning microscopy (CLSM) (Leica TCS SPE) to study the microbial attachment and biofilm formation on the sediment surfaces.

Sediment samples were collected from the designated sampling port in the microcosm using a sterile glass rod. For CLSM, a small amount of surface sediment was sampled using sterile forceps, washed with 0.9% sodium chloride solution for 5 min, and stained with 0.01% acridine orange for 20 min in a dark room. After staining, the samples were washed again with 0.9% sodium chloride solution, covered with aluminum foil, and sent to the confocal microscopy laboratory for analysis (Vidot et al., 2018).

For SEM, sediment grains were washed with Ringer solution and fixed in 2.5% glutaraldehyde for 1 h. The samples were then dehydrated using a graded ethanol series (25, 50, 75, 90, and 100%) by submerging them in each concentration for 10 min. After complete dehydration, the samples were freeze-dried, covered with aluminum foil, and transported to the SEM laboratory for analysis (Gholami et al., 2018; Inoué, 2008).

### Nucleotide sequence accession numbers

All the partial 16S rRNA gene sequences of isolates were deposited in GenBank under accession numbers OR398805, OR399151, OR399162, OR399489, and OR399515.

## Results

### The activities of methane-oxidizing bacteria in microcosm

**Figure 2:**
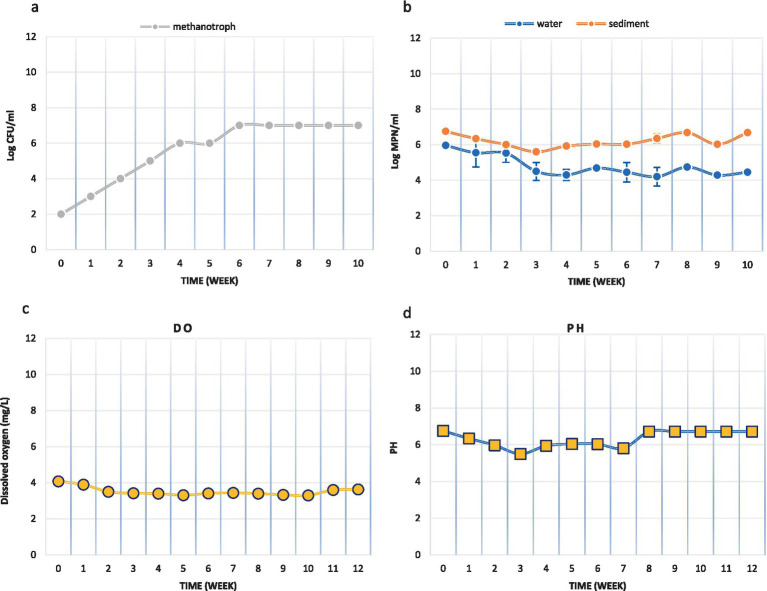
The activities of methane-oxidizing bacteria in microcosm (a) Methanotroph count (MPN/ml), (b) Microbial count (CFU/ml), (c) Dissolved oxygen concentration (mg/L) and (d) pH.

A small laboratory-scale model was designed to investigate the applicability of the MPOG^®^ technique in the Persian Gulf. The activities of methane-oxidizing bacteria on the sediment surface (0-5 cm) were studied in this model. According to Figure 2a, the population of these bacteria in the original sediment (OS) was 10^2^ MPN/ml. By continuously injecting methane gas into the system, this number increased weekly, and after 3 months, it reached 10^7^ MPN/ml. These results show that the methane-oxidizing bacteria were enriched in the microcosm during this period.

In addition, the population of heterotrophic microbial in the original water and sediment samples were 9.3 × 10^5^ CFU/mL and 5.75 × 10^6^ CFU/mL, respectively. After 3 months of methane injection, these values decreased to 4.6 × 10^4^ CFU/mL and 15.2 × 10^5^ CFU/mL, respectively (Figure 2b). So, the population of the heterotrophic microbial in the microcosm had not been changed noticeably.

During the simulation of methane gas leakage in the Persian Gulf sediments, DO and pH levels were monitored weekly. The initial DO level was 4.08 mg/L, and an effort was made to keep this level constant by aeration during the experiment. However, the DO level decreased somewhat during the experiment, which could indicate further growth of aerobic bacteria in this aerobic system. Nevertheless, the DO level reached 3.63 mg/L in the final week. The pH level remained almost constant throughout the experiment (Figures 2c,d).

### Gas chromatography analysis

In order to measure the concentration of input and output gases to the system, gas chromatography was performed before and after the experiment. At the beginning of the experiment, the input gas contained 97.37 mol% nitrogen and 2.62 mol% methane. After 90 days of continuous injection of methane into the system, the output gas from the system contained 98.24 mol% nitrogen, 1.45 mol% methane, and 0.30 mol% CO_2_ was also produced (Table 1).

**Table 1:** Measure the concentration of input and output gases to the system.

Sample	Inlet (mol%)	Outlet (mol%)
C_1_	2.6263	1.4541
N_2_	97.3737	98.2441
CO_2_	0.0000	0.3018

### Water characteristics during microcosm experiments

During the microcosm experiment, the concentrations of NO_3_^−^, _PO₄_^3−^, and SO₄^2−^ were measured. The phosphate level remained relatively constant throughout the experiment (Table 2). The initial nitrate level was 4.6 mg/L and increased to 7.4 mg/L after 90 days, indicating possible nitrification in the system.

**Table 2:** Measurement of NO3-, PO4, and SO4-2 ion concentrations during the experiment.

Ions	Original sample (ppm)	30 days (ppm)	60 days (ppm)	90 days (ppm)
NO^−^_3_	4.6	7.2	6.8	7.4
SO₄^2−^	5,044	4,923	4,828	4,670
PO₄^3−^	<0.9	<0.9	<0.9	<0.9

On the other hand, the initial sulfate level was 5,044 mg/L, but it decreased every month during the experiment, reaching 4,670 mg/L after 90 days. This decrease suggests the presence of sulfate-reducing bacteria in the microcosm, playing a role in sulfate reduction.

### Isolation of methanotrophs bacteria from microcosm

According to the results of strain isolation, a total of 5 strains containing the methane-utilizing gene were identified. Based on the 16S rRNA partial gene sequence, MT2 strain showed 99.9% similarity to *Roseibium aggregatum*. Furthermore, MT3, MT4, MT6, and MT7 strains were identified with similarities of 99.8, 98.4, 99.8, and 99.5% to *Winogradskyella poriferorum, Muricauda aquimarina, Virgibacillus kapii, and Nitratireductor aquimarinus,* respectively (Table 3).

**Table 3:** The isolated strains from microcosms during the experiment.

Strains	Top-hit taxon	Similarity (%)	Top-hit taxonomy
MT2	*Roseibium aggregatum*	99.90	*Bacteria; Proteobacteria; Alphaproteobacteria; Rhizobiales; Stappiaceae; Roseibium*
MT3	*Winogradskyella poriferorum*	99.79	*Bacteria; Bacteroidetes; Flavobacteriia; Flavobacteriales; Flavobacteriaceae; Winogradskyella*
MT4	*Muricauda aquimarina*	98.38	*Bacteria; Bacteroidetes; Flavobacteriia; Flavobacteriales; Flavobacteriaceae; Muricauda*
MT6	*Virgibacillus kapii*	99.76	*Bacteria; Firmicutes; Bacilli; Bacillales; Bacillaceae; Virgibacillus*
MT7	*Nitratireductor aquimarinus*	99.48	*Bacteria; Proteobacteria; Alphaproteobacteria; Rhizobiales; Phyllobacteriaceae; Nitratireductor*

In order to identify the methane metabolism gene in the strains, PCR was performed. The presence of a single 510 bp band in all strains (MT3, MT4, MT6, and MT7), confirmed the potential presence of the *pmoA* gene, indicating their involvement in methane metabolism. No amplification was observed in the negative control, confirming that the results were free of contamination and specific to the targeted gene.

### Microscopic analysis of microbial biofilm on the sediment surface

In addition to the planktonic water microorganisms present in the microcosm, the attached microbial population (biofilm) on sediment surfaces plays a crucial role in methane oxidation. To evaluate bacterial adhesion and biofilm thickness, SEM and CLSM analyses were conducted.

In this study, to assess the viability of microbial biofilms formed on the sediment surface, acridine orange (AO) staining was performed, followed by observation with CLSM. AO is an orange fluorescent dye that absorbs light at a wavelength of 500 nm (blue range) and emits at 526 nm (green range). This dye easily penetrates live cell membranes and binds to DNA. When bound to double-stranded DNA, AO produces green fluorescence, which can be visualized using CLSM. The microscope, by combining two-dimensional images, generates a three-dimensional view of the sample, allowing for a detailed analysis of the biofilm structure (Byvaltsev et al., 2019; Vidot et al., 2018).

**Figure 3:**
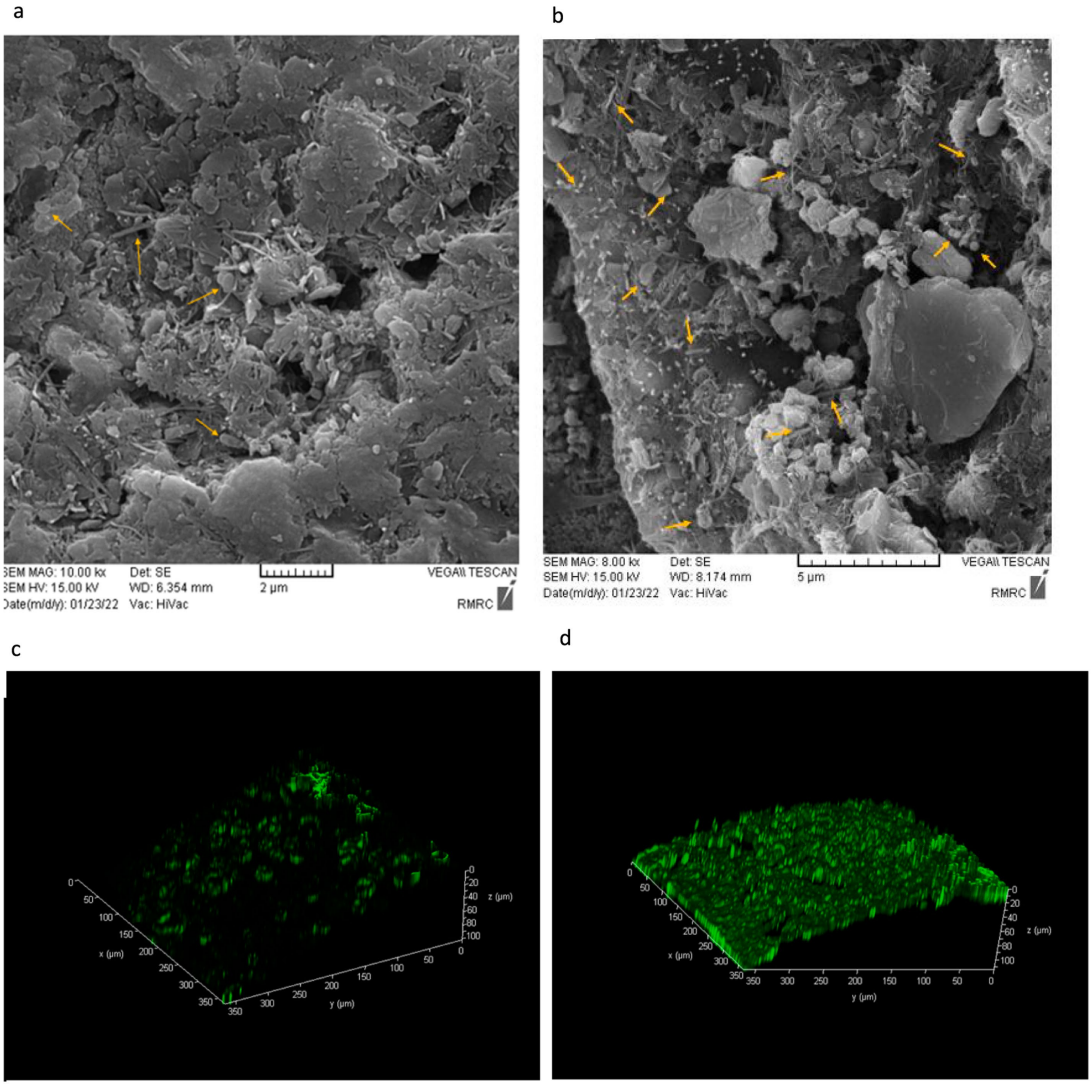
Scanning Electron Microscopy (SEM) and Acridine orange-stained confocal laser scanning microscopy (CLSM) images of biofilm thickness on the sediment surface after 3 months. (a) Original sample-SEM, (b) Microcosm sample-SEM, (c) Original sample-CLSM, (d) Microcosm sample-CLSM.

As shown in Figure 3, the original sample had fewer microorganisms and a very thin biofilm layer compared to the system sample. However, after 90 days of continuous methane injection, a much thicker biofilm layer was observed, indicated by the green fluorescence in Figure 3, suggesting an increase in the attached microbial population. In contrast, this thick green biofilm layer was not observed in the original sample. This increase suggests the presence of more methane-consuming bacteria. Therefore, based on Figure 3, a higher microbial count and a thicker biofilm layer were clearly demonstrated in the system compared to the original sample.

### Aerobic and anaerobic methanotrophs

**Figure 4:**
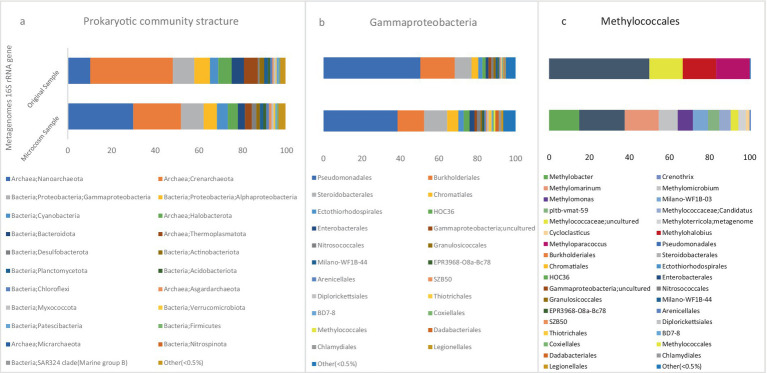
The stacked bars indicate relative abundance (x-axis%) of metagenome extracted 16S rRNA gene sequences: (a) prokaryotic phyla and Proteobacteria classes, (b) Gammaproteobacteria orders, and (c) Methylococcales genera. For (a,b) the dataset was delimited to taxonomic groups >0.5% (average of all samples). The y-axis shows the samples names.

According to the metagenome 16S rRNA data, microbial community compositions of microcosm (MS) and original sediment (OS) samples at the phylum level are visualized in Figure 4a. In the MS sample, the most abundant bacterial phylum in the population belonged to the *Proteobacteria* with a relative abundance of 16.47%. Meanwhile, the abundance of this phylum was 16% in the OS sample. There are two recognized classes for the population of methanotrophic bacteria, *Gammaproteobacteria* and *Alphaproteobacteria*. These two classes had the highest relative abundance in the bacterial population of the MS sample, accounting for 10.35 and 6%, respectively. However, this population was 9.6 and 6.5% in the OS sample, respectively.

The relative abundance of the Type I CH_4_-oxidizing taxonomic order *Methylococcales* was significantly higher in the MS than OS sample. In the MS sample, the abundance of this order was 0.7%, while in the OS sample, it was only 0.09% of the class *Gammaproteobacteria* (Figure 4b). This increase in *Methylococcales* suggests a more active role in methane oxidation within the microcosm environment due to methane injection.

**Figure 5:**
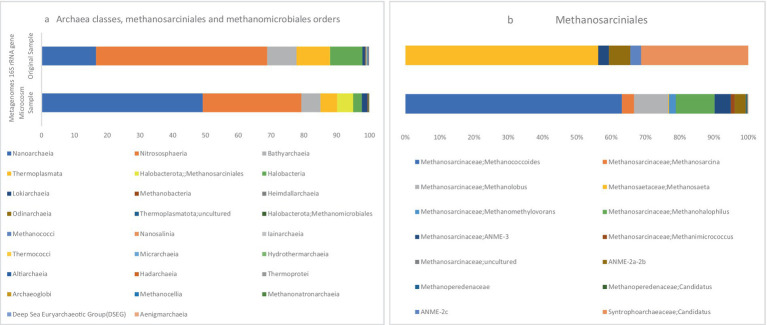
The stacked bars indicate relative abundance (x-axis%) of metagenome extracted 16S rRNA gene sequences: (a) Archaea classes, Methanosarcinales and Methanomicrobiales orders, (b) Methanosarcinales members. The y-axis shows the samples names.

Based on the 16S rRNA metagenome data, Figure 5a illustrates the visualization of archaeal orders *Methanosarcinales* and *Methanomicrobiales* in MS and OS samples. In the MS sample, the relative abundances of *Methanosarcinales* and *Methanomicrobiales* within the total archaeal population were 4.86 and 0.04%, respectively, indicating a notable presence of these archaea. In contrast, these two orders were present in minor amounts in the OS sample, suggesting a significant shift in the archaeal community in response to the microcosm conditions. ANME-2 and ANME-3 are clustered within the order *Methanosarcinales*, while ANME-1 belongs to a new order that is distantly related to the orders *Methanosarcinales* and *Methanomicrobiales* (Bhattarai et al., 2019).

Overall, in contrast to the MS, the OS, exhibited microbial community characteristics indicative of a more stable and undisturbed environment. While the relative abundance of methanotrophic bacteria and methane-oxidizing taxa such as *Methylococcales* was lower in OS compared to MS, this reflects the natural baseline of the sediment without external methane injection. This lower presence of key methanotrophic groups in OS, suggests that the native microbial community was less active in methane oxidation under ambient conditions. This highlights the importance of environmental factors, such as methane availability, in shaping microbial community dynamics, as the significant increase observed in MS was directly related to the introduction of methane into the system.

## Discussion

### Microcosm experiments

A laboratory model was used to simulate methane leakage in the sediments of the Persian Gulf, using the MPOG^®^ technique to facilitate methane migration on the sediment surface. Continuous methane injection enriched Methane-Oxidizing Bacteria on the sediment surface.

Based on the obtained results, five strains were isolated from the microcosm. Strain MT2 is associated with members of the genus *Roseibium* and shares 99% similarity with the species *aggregatum*. Various species of the genus *Roseibium* have been reported in different studies. In 2014, a new species named *aquae* was isolated from a lake with 3% salinity (Zhong et al., 2014). Additionally, in 2017, a species named *sediminis* was separated from surface sediments in the Bohai Sea (Liu et al., 2017b). According to the conducted studies, strain 161A was isolated from surface lake water (BacDive, n.d.). Based to the KEGG database, *Roseibium* possesses genes involved in formaldehyde assimilation in the methane metabolism pathway (KEGG PATHWAY Database, 2023a; Yurimoto et al., 2005). However, no reports have been made regarding the gene *pmoA* in this bacterium.

Strain MT6 exhibited 99.76% similarity to the species *kapii* from the genus *Virgibacillus*. According to the KEGG database, *Virgibacillus* sp. SK37 harbors genes involved in formaldehyde assimilation in the methane metabolism pathway (KEGG PATHWAY Database, 2023b). However, there is no available data regarding the methane metabolism in other species of this genus, namely *kapii*. *Muricauda aquimarina*, is a bacterium that exhibits a preference for slightly halophilic conditions. It was discovered and isolated from a salt lake in close proximity to the Hwajinpo beach in Korea (Yoon et al., 2005).

As mentioned earlier, strain MT3 shows 99.79% similarity to *Winogradskyella poriferorum* based on the results obtained in this study. The growth of *W. poriferorum* (the type strain) is strictly aerobic, occurring between 12 and 44°C and within a pH range of 6.0 to 10.0, with a requirement for NaCl (1.0–4.0%) for growth (Lau et al., 2005). Notably, *Winogradskyella* has been identified as a bacterium capable of degrading organic compounds, particularly in environments rich in hydrocarbons, such as oil. This role is attributed to its ability to utilize complex organic compounds as energy sources, as demonstrated in several studies on marine environments (Liu et al., 2017a). In our methane-enriched experimental system, the isolation of a *Winogradskyella* strain may indicate conditions similar to those in oil-contaminated environments, where this bacterium plays a role in breaking down organic compounds or potentially regulating ecological conditions. Its presence in our system suggests its possible involvement in metabolizing organic compounds related to methane or hydrocarbons.

Thus, the connection between *Winogradskyella*’s known presence in hydrocarbon-rich environments, such as oil spills, and its occurrence in our methane-enriched system highlights its potential role in the breakdown of organic matter associated with methane. This finding also implies a broader involvement of *Winogradskyella* in carbon metabolism and biogeochemical cycles in aquatic ecosystems.

In this study, we isolated strain MT4 from our system, which shares 98.38% similarity with *Muricauda aquimarina*. Although there is no direct evidence linking this bacterium to methane degradation, previous research has highlighted *Muricauda’s* significant role in organic matter degradation and nutrient cycling (Meora et al., 2024). These findings suggest that *Muricauda* in our system may contribute to the breakdown of organic compounds, potentially including those associated with methane, indicating its relevance in critical biogeochemical processes.

We isolated a strain of *Nitratireductor aquimarinus* (MT7) from our system, a bacterium known for its role in the nitrogen cycle, specifically in nitrate reduction processes. Although no direct evidence links *N. aquimarinus* to methane degradation, species within the *Nitratireductor* genus are well-recognized for their ability to degrade organic compounds in aquatic environments. These bacteria often play a significant role in the degradation of organic matter and contribute to nutrient cycling. Therefore, *N. aquimarinus* in our system could potentially aid in the breakdown of complex organic compounds, supporting key biogeochemical processes such as carbon and nitrogen metabolism. Furthermore, a recent study identified a novel species within the *Nitratireductor* genus, *Nitratireductor thuwali* sp. *nov*., isolated from the extreme and oligotrophic conditions of Red Sea mangrove sediments. This species has been shown to metabolize various organic compounds, including straight-chain alkanes and organic acids, under aerobic, heterotrophic conditions. These findings highlight the potential versatility of *Nitratireductor* species in breaking down diverse carbon substrates, suggesting that *N. aquimarinus* in our system may exhibit similar capabilities, despite the lack of direct evidence linking it to methane degradation (Marasco et al., 2023; Jang et al., 2011).

The results indicated that five specific isolates (MT2, MT3, MT4, MT6, and MT7) were able to utilize methane as their sole source of carbon for growth and survival. These findings have important implications for further research and understanding of the ecological roles and metabolic capabilities of these microorganisms.

According to the water characteristics during microcosm experiments, the observed increase in nitrate levels during the experiment indicates the activity of nitrifying bacteria, converting nitrite to nitrate, which is an essential process in the nitrogen cycle. The presence of *Nitrobacter*, *Nitrospina*, and *Nitrospinota* supports this conclusion, as they are known to be involved in nitrite oxidation and nitrification in different environments.

The decrease in sulfate levels over time suggests the presence of sulfate-reducing bacteria, contributing to sulfate reduction. This process is vital in various ecosystems, as it helps in the cycling of sulfur compounds and plays a significant role in anaerobic environments.

Overall, the results of the microcosm experiment demonstrate the dynamic interactions between different microbial communities and their impact on the concentrations of nitrate and sulfate in the system. These findings contribute to our understanding of nutrient cycling and microbial activities in aquatic environments.

According to the gas chromatography analysis, the decrease in methane gas could indicate that methane-consuming bacteria have grown and used methane as a source of carbon and energy. Additionally, the production of CO2 gas in the output of the system indicates the activity of microbes and microbial respiration.

The increase in the attached microbial population and the formation of a thick biofilm layer after continuous methane injection support the enrichment of Methane-Oxidizing Bacteria in the microcosm during the experiment. The observed growth of methane-consuming bacterial populations in the system suggests that the microorganisms have utilized methane as a source of carbon and energy, leading to the production of a thick biofilm. In fact, the results from SEM and CLSM analyses further highlight the importance of biofilm in methane oxidation. Presence of a thicker biofilm layer in the system sample indicates that the attached microbial community on the sediment surface plays a crucial role in methane consumption and microbial respiration, leading to the production of CO_2_ gas as observed in the gas chromatography analysis.

In summary, these findings demonstrate the significance of the biofilm community in methane metabolism and its potential impact on the carbon cycle in the Persian Gulf sediment. Understanding the dynamics of microbial biofilms and their response to methane exposure can provide valuable insights into the ecological roles of methane-oxidizing bacteria and their contribution to greenhouse gas regulation in marine environments. Further research in this area may lead to better management strategies for mitigating methane emissions and their potential effects on the global climate.

### Microbial community analysis

#### Aerobic methanotrophs in sediment

Based on the results, further analysis of the order *Methylococcales* revealed that the CH_4_ oxidizers were comprised of two bacterial families: *Methylomonadaceae* and *Methylococcaceae*. The relative abundances of these two families in the microbial population were 0.2 and 0.01%, respectively. However, these families were observed in much lower numbers in the OS sample.

**Figure 6:**
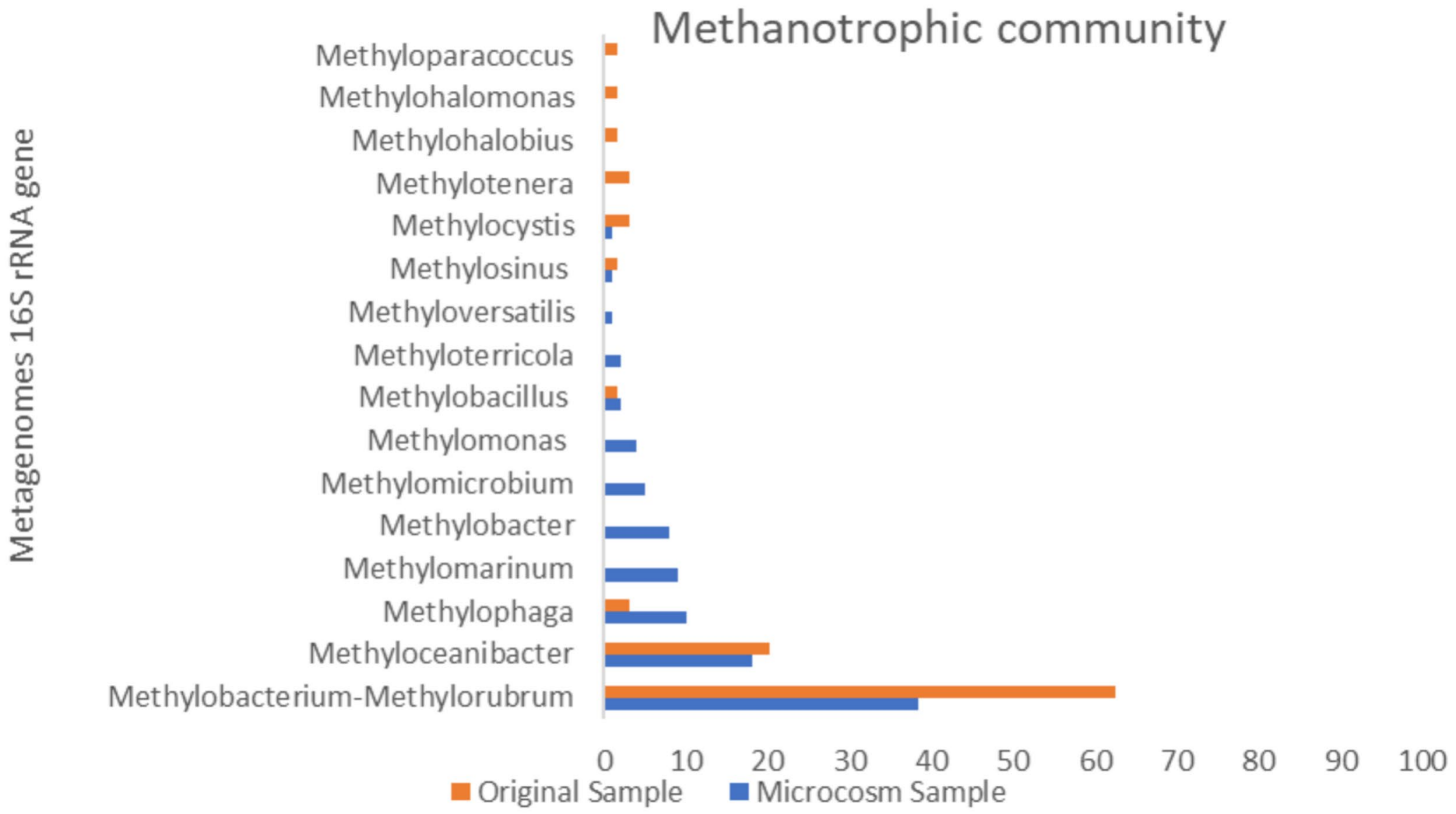
Comparison of methanotrophic population in original sample and microcosm samples.

The taxonomic classification of all metagenomic sequences revealed that *Methylococcales* had a higher relative abundance of reads in the MS sample, Figure 4c. The majority of identified methanotrophic genera in the MS sample were classified under the family *Methylomonadaceae*, including the most dominant genus, *Methylobacter* (8%), *Methylomarinum* (9.78%), *Methylomicrobium* (5.43%) and *Methylomonas* (4.34%) of methanotrophic population Figure 6. Additionally, the identified methanotrophic genera belonging to the family *Methylococcaceae* included the genus *Methyloterricola* (2%). None of the genera belonging to these families were detected in the OS sample. However, two methanotrophic species belonging to *Methylococcales*, named *Methyloparacoccus* and *Methylohalobius*, were observed in the OS sample. Furthermore, the majority of the sequences belonging to *Methylococcales* were assigned to the genus *Methylomarinum*, which is an obligate aerobic methanotroph capable of utilizing only the particulate methane monooxygenase (pMMO) pathway. This bacterium has a marine habitat and requires 2–3% salinity for growth. Its optimal growth temperature is 37°C. It grows on methane and methanol and assimilates C1 compounds via the ribulose monophosphate pathway (Hirayama et al., 2013). Due to the salinity of the water in the Persian Gulf being 3% (Rezaei Somee et al., 2021), and its growth conditions, this genus was one of the most dominant genera in the microcosm.

*Methylobacter*, a genus of *Gammaproteobacteria*, is the predominant and highly active group of methanotrophs in various ecosystems such as peat soils, tundra, ponds, lakes, sub-glacial sediments, lake sediments, rice paddies, and landfills. These environments, known as the largest methane producers in the world, have high concentrations of dissolved methane. This implies that methylotrophic bacteria are typically exposed to such high concentrations of methane and have likely developed adaptations to effectively assimilate and thrive in such environments (Tveit et al., 2023). This genus was not observed in the OS sample.

The other dominant genus in the system is *Methylophage* bacterial (*Nitrosococcales* order), which accounts for a relative abundance of 10% of the methanotrophic population. The relative abundance of this order in the OS sample was very low (0.03%). The genus *Methylophaga* comprises members of the *Gammaproteobacteria* that are halophilic and exhibit methylotrophic properties. These bacteria have been isolated from marine environments. These species are capable of growing under denitrifying conditions in the presence of nitrate and methanol, which makes them significant in nitrogen cycling processes in the ocean (Wei et al., 2022; Villeneuve et al., 2012). Because of the high salinity of the Persian Gulf water, this halophilic bacterium had become prevalent in the microcosm.

Microbial population analysis in Haakon Mosby Mud Volcano demonstrated that the high availability of methane has led to the abundance of aerobic methanotrophic genera (*Methylobacter* and *Methylophaga*) as well as anaerobic methanotrophs (mostly ANME-3 lineage) (Lösekann et al., 2007).

Some members of the *Rhizobiales* order, which belong to the class *Alphaproteobacteria*, have been identified as aerobic methanotrophs. The family *Beijerinckiaceae*, which belongs to this order, was observed in the MS sample. The genera *Methylocystis* and *Methylosinus* belonging to this family, were found to be extremely rare in the microcosm, indicating their low abundance. Also, the results of the metagenome 16S rRNA data showed the presence of the genus *Methylobacterium*-*Methylorubrum* (family *Beijerinckiaceae*) which is a facultative methylotrophs bacterium, in the OS sample. This genus was also observed in the MS sample, however, its abundance remained unchanged throughout the experiment. As the pMMO enzyme is a copper-containing enzyme, a low copper-to-biomass ratio can initiate the expression of sMMO in some methanotrophs of the family *Beijerinckiaceae* that only encode sMMO (Kalyuzhnaya et al., 2020). Therefore, the presence of sufficient amounts of copper in the microcosm may have restricted the growth of the family *Beijerinckiaceae*. The studies have shown that the concentration of copper ions in the water and sediments of the Asaluyeh are 1.4 ppm (depth of 5 meters) and 11 ppm (depth of 20 meters) respectively (Rezaei Somee et al., 2021).

Methane oxidation in the studied environment can be facilitated by methylotrophs, which have the capability to utilize C1 substrates as their sole source of energy and carbon (Singh et al., 2022). It is worth noting that methylotrophs often coexist with methanotrophs and play a significant role in the overall methane oxidation process (Takeuchi et al., 2019) family *Methyloligellaceae* members within the *Rhizobiales* order were detected in the MS sample with high abundance. These microorganisms exhibit the ability to utilize both methylated compounds and methane as energy and carbon sources (Takeuchi et al., 2019). These findings highlight the potential contribution of methylotrophs, particularly those from the family *Methyloligellaceae*, to the overall microbial dynamics and methane oxidation process in the studied environment.

Another recognized class of aerobic methanotrophs is *Verrucomicrobiae* which belong to *Verrucomicrobiota*. None of the methanotrophic genera within this class were observed in the samples.

The study conducted on the microbial population in Shallow Boreal Coastal Zones revealed that the majority of methanotrophs belonged to the order *Methylococcales*. The genus *Methyloprofundus* was identified as the most predominant genus within this order (Broman et al., 2020).

Based on the results obtained, the dominant microbial pattern consisted of methanotrophic bacterial lineages, following continuous injection of methane into the system, comprising genera *Methylobacter*, *Methylomarinum*, *Methylomicrobium*, *Methylomonas*, and *Methylophage*. Most of the taxa within these genera were comprised of species that had not been classified at this taxonomic level. The high abundance of these genera in the system indicates their significant role in aerobic methane oxidation in the sediments of the Persian Gulf. In fact, this microbial pattern emerges as methane migrates from reservoirs and reaches the sediment surface. Therefore, identifying this microbial population anomaly in the Persian Gulf can provide valuable insights for pre-drilling gas reservoir exploration.

#### Anaerobic methanotrophs in sediment

Based on the results, the ANME-3 clade, which belongs to the family *Methanosarcinaceae*, constituted 4.74% of the *Methanosarcinales* abundance. Additionally, this order comprised 3.20% ANME-2a/b and ANME-2c 0.19%, respectively, Figure 5b. In a study conducted in 2021 on microbial populations in the sediments of the northern part of the Barents Sea, ANME-2a/b and ANME-3 were found to be the predominant archaeal clades at depths of 0–5 cm (Begmatov et al., 2021). In marine sediment ecosystems, the distribution of ANME clades is characterized by zonation. Specifically, ANME-2a/b clades dominate the upper layers, while deeper zones show an increased abundance of ANME-2c and/or ANME-1. This zonation pattern reflects a niche differentiation among the ANME clades, indicating their adaptation to specific ecological niches (Timmers et al., 2015b; Timmers et al., 2017).

In the MS sample, sulfate-reducing bacteria belonging to the *Desulfobulbus* were detected among the sulfate reducers. However, their presence was found to be characterized by a notably low relative abundance. Previous studies have frequently reported the association of *Desulfobulbus* with ANME-3 (Lösekann et al., 2007; Niemann et al., 2006). The results of this study align with previous reports suggesting the existence of independent AOM apart from ANME-3 (Bhattarai et al., 2017). ANME-3 is often found in cold seep areas and mud volcanoes with high CH_4_ partial pressures and relatively low temperatures (Vigneron et al., 2013). Despite this, studies have shown the effect of gas pressure on the formation of ANME-SRB consortia. At a pressure of 20 MPa, ANME-3 and *Desulfobulbus* were observed in fewer numbers and mostly formed small clusters of ANME-3/*Desulfobulbus*. At higher pressures of methane flux, 40 MPa, this consortium did not form. The results indicate that this consortium was mostly formed at a pressure of 20 MPa, suggesting that these bacteria likely have mutual benefits at this pressure (Cassarini et al., 2019). Therefore, it can be hypothesized that this consortium has not been formed due to the absence of pressure in the designed system, leading to independent anaerobic methane oxidation by ANME-3 clade.

In the MS sample, sulfate-reducing *delta-proteobacteria* (phylum *Desulfobacterota* in genome-based taxonomy) were found with a relative abundance of 2%. In contrast, the abundance of this phylum was 0.1% in the OS sample. SEEP-SRB1 (family *Desulfosarcinaceae*) was the dominant group among SRB, and there was a relatively low abundance of SEEP-SRB4 (*Desulfocapsaceae*). ANME-1, SEEPSRB2, and SEEP-SRB3 were not detected in the MS sample. The analysis results showed that in the MS sample, the relative abundance of SEEP-SRB1 and SEEP-SRB4 in the microbial population was 4 and 0.02%, respectively. The observed coexistence of ANME-2a/b and SEEP-SRB1 groups strongly supports the proposition that AOM is linked to sulfate reduction. This association is consistently demonstrated by data obtained from an enrichment culture comprising ANME-2a/b and SEEP-SRB1 sulfate-reducing microorganisms (Timmers et al., 2015a; Schreiber et al., 2010).

The research conducted on the microbial community in Jiaolong, an active methane seep site located in the northern continental slope of the South China Sea (SCS), revealed interesting findings. The predominant microorganisms identified were SEEP-SRB1, which were coupled with ANME-2 and ANME-3. Additionally, a small population of SEEP-SRB4 was observed, also coupled with ANME-2 and ANME-3. However, ANME-1, SEEP-SRB2, and SEEP-SRB3 were notably absent in the sediment samples collected during the study (Li et al., 2020). These findings shed light on the specific microbial composition and interactions in this methane-rich environment, contributing to our understanding of the microbial ecology in such seep sites.

The observed reduction in sulfate levels within the system after 90 days period may be ascribed to the metabolic activity of these sulfate-reducing bacteria. In the investigated microcosm, the presence of anaerobic methanotrophs from the family *Methylomirabilaceae*, specifically nitrite-reducing bacteria (Ettwig et al., 2010), was not observed.

This observation suggests that the oxidation of methane in the sediment surface is primarily conducted through aerobic pathways rather than anaerobic processes.

According to MPOG^®^, the presence of a relatively impermeable layer at the top of a reservoir is one of the conditions for the formation of an oil and gas accumulation. This layer, known as the cap rock, is usually not completely impermeable, and there is a possibility of gradual escape of small amounts of hydrocarbons or other gases through leaks and fractures, which can be intensified over time (Rice, 2022). This migration and escape typically occur vertically and upward (Schumacher, 2019). However, the rate of methane migration to the surface is approximately 0.6–2 m/day (Rice et al., 2002). Buoyancy theory has been recognized as an important mechanism for the vertical migration of gases from the reservoir to the surface. In this case, gas escapes from the cap rock. Gas bubbles have buoyancy contrary to gravity. Nevertheless, the numerous rock layers along the migration path to the surface alter the gas migration pathway. This holds true for marine environments (Klusman and Saeed, 1996; Schumacher and Abrams, 1996). In fact, the faults and fractures created over time lead to the formation of passages at the top of the reservoir that are saturated with water. When the reservoir pressure exceeds the pressure of water in these passages, gas can escape (Rice, 2022).

In this study, considering continuous air supply from the surface and injection of the methane-air gas mixture from the bottom into the system, aerobic and anaerobic methane-oxidizing microbial populations were observed in the simulated system. These methane-oxidizing archaea (although in low numbers) were present in the 0–5 cm depth of the sediment in the system. Given the presence of sedimentary rock layers, approximately 10 centimeters in thickness within this simulation, gas molecules undergo collisions with these layers, resulting in changes in their direction. Consequently, localized anaerobic conditions may be established within certain regions of the system. Therefore, anaerobic conditions may have been established in certain locations of the system.

In summary, our scientific investigation involved simulating methane gas leakage in the sediments of the Persian Gulf through the utilization of a laboratory model. The employed methodology was centered around the MPOG^®^ technique, which facilitated the migration of methane on the sediment surface. Through the consistent injection of methane into the model, we successfully enriched Methane-Oxidizing Bacteria on the sediment surface. By analyzing the microbial population present in the sediment using NGS, we were able to identify a distinct pattern of microbial population that exhibited the ability to consume methane gas within the sediments of the Persian Gulf. Based on the 16S rRNA sequencing dataset, the bacterial community pattern of methanotrophs consists of genera *Methylobacter, Methylomarinum, Methylomicrobium*, *Methylomonas*, and *Methylophage*.

The anaerobic methane-consuming community comprised the ANME-2 and ANME-3 clades. This suggests that these microbes are capable of coexisting and thriving in the sediment of the Persian Gulf when exposed to long-term methane exposure.

Identifying this pattern, as biological markers, alongside other geophysical and geological data in the Persian Gulf can increase the success rate of gas reservoirs exploration. By utilizing this microbial pattern, it is possible to determine better drilling locations, prevent unsuccessful drilling attempts, and shorten the exploration timeframe.

It is worth noting that these results demonstrate the specific characteristics of the microbial community in Persian Gulf sediments within a laboratory model, and they may not apply to other environments and microbial communities.

Further studies involving functional gene analysis will provide more comprehensive information on microbial activity and metabolic pathways present in this community.

## Data Availability

The data presented in the study are deposited in NCBI/SRA repository, accession number PRJNA1004775.
